# A Commentary on the Role of Pulmonary Function Parameters in Idiopathic Pulmonary Fibrosis Follow‐Up

**DOI:** 10.1111/crj.13797

**Published:** 2024-06-23

**Authors:** Guliz Degirmenci, Celal Satici

**Affiliations:** ^1^ Department of Pulmonology Health Science University, Yedikule Chest Diseases and Thoracic Surgery Training and Research Hospital Istanbul Turkey

**Keywords:** antifibrotic treatment, diffusing lung carbon monoxide, forced vital capacity, idiopathic pulmonary fibrosis

Idiopathic pulmonary fibrosis (IPF) is a progressive disease and has a high mortality rates [1]. While the overall prognosis of IPF is poor, the decline in pulmonary function tests and progression to death varies widely [2]. Identification of patients with slow progression or rapid progression would be valuable for subsequent studies, including genetic analyses and investigation of IPF clinical phenotypes. Additionally, it could influence treatment decisions, including the timing of transplantation. We congratulate Doubkova and colleagues for their study aiming to establish the impact of demographic parameters, pulmonary function parameters and high‐resolution computed tomography scores [1]. It was revealed that diffusing lung carbon monoxide (DLCO) decline seems to be superior to forced vital capacity (FVC) decline in predicting mortality. After a thorough review of their article, we offer the following comments to enrich the understanding of their study.

FVC is the most common outcome used in investigating the course of patients with IPF and change in FVC percentage predicted over time is a well‐known predictor of mortality [3]. The primary endpoint as FVC decline in INPULSIS [4], ASCEND [5], CAPACITY [6] and TOMORROW [7] studies. However, DLCO decline was found to be an independent predictor of mortality after multivariate analysis instead of FVC decline in this study [1]. Statistically, only more clinically significant parameters should be included in the multivariate analysis among those showing high correlation. In line with this, it should be noted that both FVC and DLCO decline, which are expected to have a high correlation, were included in the multivariate analysis. This may lead to a multicollinearity problem and impact the accuracy of the results. To address this issue, it may be beneficial to create two separate models [Model 1: FVC model (not including DLCO) and Model 2: DLCO model (not including FVC)].

Regarding baseline characteristics, there were significant differences in the extent of lung involvement, FVC (%) and DLCO (%) found between alive and deceased patients. Deceased patients were more likely to have higher alveolar and interstitial scores, as well as lower DLCO (%) and FVC (%). Since baseline FVC (%) and DLCO (%) were not included in the univariate Cox analysis, we cannot determine the independent effect of decline in FVC (%) and DLCO (%). This is important because patients with more severe baseline conditions are more likely to deteriorate and experience mortality.

As authors indicated, pulmonary hypertension has not been assessed. However, this may affect the prognostic role of DLCO (%), which is the primary conclusion of this study, because DLCO is expected to be significantly low in patients with pulmonary hypertension. Indeed, given the highly variable clinical course of IPF, there are also significant differences in treatment response among patients. Therefore, the absence of data on antifibrotic treatment could potentially impact the interpretation of the findings.

## Author Contributions

Guliz Degirmenci and Celal Satici contributed to the design and implementation of the study, evaluation of the analysis of the results and writing of the article.

## Conflict of Interest

The authors declare no conflicts of interest.

## Data Availability

Data sharing is not applicable to this article as no datasets were generated or analysed during the current study.
